# Clopidogrel in Orthopaedic patients: a review of current practice in Scotland

**DOI:** 10.1186/1477-9560-5-6

**Published:** 2007-05-25

**Authors:** Jibu J Joseph, Anand Pillai, Diane Bramley

**Affiliations:** 1 Department of Orthopaedic Surgery, Southern General Hospital, 1345 Govan Road, Glasgow, Glasgow, G51 4TF, Scotland, UK; 2 Department of Orthopaedic Surgery, Monklands Hospital, Monkscourt Avenue, Airdrie, ML6 0JS, Scotland, UK; 3 Consultant Orthopaedic Surgeon, Department of Orthopaedic Surgery, Monklands Hospital, Monkscourt Avenue, Airdrie, ML6 0JS, Scotland, UK

## Abstract

**Background:**

Clopidogrel bisulfate is an antiplatelet agent used to prevent ischaemic events in patients with vascular disease. Current guidelines recommend withholding clopidogrel for 7 days pre-operatively. However these are not based on orthopaedic patients. We therefore decided to survey current orthopaedic practice to see whether this complied with available clinical data.

**Method:**

A questionnaire was sent to all orthopaedic consultants in Scotland.

Four haematology departments, and the manufacturers, were contacted to ask for their recommendations, and a database search was performed.

**Results:**

140 questionnaires were sent with a 60.7% response. 84.7% of respondents have encountered patients on clopidogrel. Of those, 13.9% did not routinely stop it, and 86.1% stopped it 5–21 days pre-operatively (47.2% at 7 days).

45.9% had a unit policy on stopping clopidogrel, and the majority (69.4%) did not consult their haematology department prior to instituting their policy.

Increased peri-operative bleeding was the most reported complication (22.6%). However this was only noted in those who stopped clopidogrel greater-than 7 days pre-operatively.

Haematology advice ranged from continuing clopidogrel peri-operatively to stopping it 7 days pre-operatively and starting low-molecular-weight-heparin for thrombo-prophylaxis. The manufacturers suggested stopping clopidogrel 7 days pre-operatively. An internet search did not reveal any data on the effect of clopidogrel peri-operatively in orthopaedic patients.

**Discussion:**

Recommendations on stopping clopidogrel have evolved from studies conducted on patients undergoing cardio-thoracic surgery. There is no data available on the effect of clopidogrel in orthopaedic practice. Our survey indicates that increased bleeding has not been found in patients who continue clopidogrel peri-operatively.

Almost half of respondents complied with current recommendations, stopping clopidogrel 7 days pre-operatively. However there remains a lack of consensus amongst orthopaedic surgeons.

Currently elective patients should stop clopidogrel 7 days pre-operatively, and emergency patients should stop clopidogrel on admission, however their operation should not be delayed due to clopidogrel usage.

## Background

Clopidogrel bisulfate (Plavix^®^) is an antiplatelet agent that is used in the secondary prevention of ischaemic events in patients with ischaemic heart disease or peripheral vascular disease that are intolerant of aspirin, or in those who require additional antiplatelet therapy in combination with aspirin [1].

Atherosclerotic disease of both peripheral and central arteries is extremely common in the British population [2]. Combination therapy with statins and antiplatelet agents such as aspirin and clopidogrel is increasingly being used to combat the morbidity and mortality associated with these conditions [3]. Therefore, it is probable that more patients presenting to hospital for elective or emergency orthopaedic surgery will be on an antiplatelet agent. For patients taking the antiplatelet agent aspirin, current Scottish Intercollegiate Guidelines Network (SIGN) recommendations state that aspirin can be continued peri-operatively and used as deep venous thrombosis (DVT) prophylaxis [4]. There is however no SIGN recommendation for patients taking clopidogrel.

We have audited the current orthopaedic practice in Scotland with regards to patients taking clopidogrel admitted both in the trauma and elective setting. Our aim in this study was to compare current practice with evidence based recommendations, the pharmaceutical industry advice, and haematology advice, in order to ascertain whether or not there was a consensus. We highlight the need to develop a uniform protocol based on current best evidence in dealing with these patients.

## Methods

**Figure 1:**
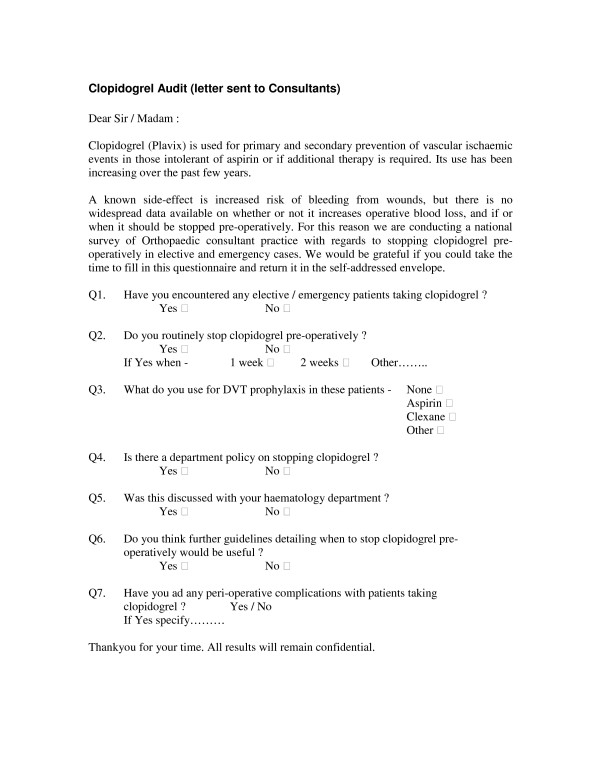
Clopidogrel Audit Letter.

A standardised anonymous questionnaire was sent to all practising orthopaedic consultants in Scotland (Fig 1). They were requested to return the completed questionnaire to the first author. The haematology departments at all four Scottish university teaching hospitals (Edinburgh Royal Infirmary (ERI), Glasgow Royal Infirmary (GRI), Aberdeen Royal Infirmary (ARI), and Dundee Ninewells Hospital), were also contacted to establish whether they had any advice regarding management of orthopaedic patients taking clopidogrel.

The pharmaceutical company responsible for manufacturing Plavix ^® ^(Bristol-Myers Squibb) was contacted to see whether they had any recommendations specific to orthopaedic patients, and to ascertain if any research had been conducted in order to support their recommendations. The current British National Formulary (BNF 51) recommendation was also noted, and a review of available literature was performed through google, pub med, medline, and Cochrane database searches.

## Results

One hundred and forty questionnaires were sent, with a 60.7% response rate (85 replies). 84.7% of respondents had encountered patients on clopidogrel in their routine practice. There was wide variation in management between different respondents. Almost half (47.2%) stopped clopidogrel 7 days pre-operatively (Table 1), while 13.9% did not routinely stop the medication. The remaining 38.9% stopped the medication at a variable interval between 5 days and 21 days before surgery. No comment was made as to whether or not the decision to stop clopidogrel, and if so when, was based on urgency of surgery (ie. trauma or elective procedures). Two consultants who had not personally encountered patients on clopidogrel said they would ideally discontinue the medication 7 days pre-operatively.

**Table 1:** When do you routinely stop clopidogrel

**When Do You Stop Clopidogrel**	**Number (%)**
Do not stop	10 (13.9%)
5 days	1 (1.4%)
7 days	34 (47.2%)
10 days	4 (5.6%)
14 days	17 (23.6%)
21 days	1 (1.4%)
No time given	5 (6.9%)

Increased peri-operative complications were reported by more than one quarter (25.8%) of respondents (Table 2), the most common of which was increased intra-operative and post-operative bleeding (22.6%). Less than half of respondents (45.9%) stated that there was a unit policy in place on whether or not clopidogrel should be stopped. In addition, the majority (69.4%) did not seek specific haematology advice prior to instituting their policy on stopping the medication. The vast majority (70.6%) felt further guidelines would be useful.

**Table 2:** Perioperative complications in patients taking clopidogrel

**Do you routinely stop clopigorel**	**Have you found increased peri-operative complications in patients taking clopidogrel**	**Complications encountered**
Yes (62)	Yes – 16 (25.8%)	Bleeding – 14 (22.6%) Haematoma – 2 (3.2%) Wound infection – 2 (3.2%) CVE * – 1 (1.6%)
	No – 46 (74.2%)	N/A
No (10)	No – 10 (100%)	N/A

* CVE – Cerebro-Vascular Event

We obtained recommendations from all four Scottish university teaching hospital haematology departments regarding the peri-operative management of orthopaedic patients taking clopidogrel (Table 3). All four haematology departments suggested that a cardiology opinion should be sought prior to altering medication, and that the decision whether or not to stop clopidogrel depends on the balance between risks of bleeding compared to the risk of an ischemic event. Most haematologists felt that clopidogrel should ideally be stopped before planned surgery and that if additional DVT prophylaxis was needed (high risk patients), low molecular weight heparin (LMWH) or fondaparinux should be used. Discontinuing clopidogrel, and commencing these patients on aspirin (as many orthopaedic units use low dose aspirin as their standard DVT prophylaxis) was not thought to be advisable, as this does not allow platelet function to recover. For those taking aspirin and clopidogrel together, it was suggested that aspirin could be continued as DVT prophylaxis if indicated once clopidogrel was stopped. In the trauma setting, there was a difference of opinion as to what the best practice should be with suggestions that it may be possible to continue clopidogrel during the peri-operative period utilising its anti-platelet action for thrombo-prophylaxis.

**Table 3:** Haematology Advice

**Haematology Department**	**Should clopidogrel be stopped**	**If stopped when**	**What DVT prophylaxis should be used**
GRI	No (if not on aspirin)	N/A	Clopidogrel
	Yes (if on aspirin also)	7 days	Aspirin
ERI	Yes	2 – 3 days	LMWH *
ARI	Yes	3 days	Fondaparinux if high risk otherwise none
Ninewells	Yes	7 – 10 days	LMWH if high risk otherwise none

* LMWH – Low Molecular Weight Heparin (eg. Enoxaparin/Clexane)

The manufacturers of clopidogrel, Bristol-Myers Squibb (UK), were of the opinion that if an antiplatelet effect is not desired, clopidogrel should be stopped 7 days in advance of surgery. However no studies were undertaken specifically on orthopaedic patients. The current volume of the BNF states that clopidogrel should be stopped 7 days pre-operatively [5]. There was no specific advice for orthopaedic patients. Literature review did not reveal any published data on the effect of continuing or discontinuing clopidogrel in orthopaedic elective and trauma patients. There was also no data available on using clopidogrel as peri-operative DVT prophylaxis in orthopaedic patients.

## Discussion

Clopidogrel is an antiplatelet agent that is being increasingly prescribed in both general practice and hospital medicine due to its synergistic action with aspirin, and also for those intolerant of aspirin. The mechanism of action is by irreversibly inhibiting binding of adenosine diphosphate (ADP) to its platelet receptor. This inhibits ADP-mediated activation of glycoprotein IIb/IIIa complex. This in turn inhibits platelet aggregation [1]. As the receptor modification is irreversible, platelet aggregation is impaired for the remainder of the platelets lifespan, generally 7 days. It is because of this permanent effect that the manufacturers, and the current BNF guidelines, recommend stopping clopidogrel 7 days in advance of elective surgery if the antiplatelet effect is not desired [5]. Therefore current practice for the majority reflects the current recommendation.

This recommendation however was based on studies that showed increased rate of post-operative bleeding, need for transfusion, and re-operation rate in patients undergoing cardiothoracic surgery that had clopidogrel continued pre-operatively [6-9]. To our knowledge there has been no research into continued use of clopidogrel peri-operatively in orthopaedic patients.

Patients undergoing orthopaedic surgery have a high incidence of thrombo-embolic complications [10], especially if no DVT prophylaxis is used [11]. DVT prophylaxis can be both mechanical and pharmacological, and both methods have been shown to decrease thrombo-embolic phenomena in orthopaedic patients [12-14]. Although the new National Institute for Clinical Excellence (NICE) guidelines do not mention it [15], the Scottish Intercollegiate Guidelines Network (SIGN guidelines) suggests that one method of DVT prophylaxis is aspirin [4]. This was also supported by one of the haematology departments we contacted. It has been shown that aspirin reduces the risk of thrombo-embolic disease, and that the risk of increased postoperative bleeding, haematoma, or infection is low [13]. Therefore we pose the question that although the mechanism of action of both aspirin and clopidogrel is different (aspirin inhibits the cyclo-oxygenase dependent production of thromboxane A2); if aspirin has been shown to be a safe and effective method of DVT prophylaxis in orthopaedic patients could clopidogrel not be used in patients already taking the medication? Currently there appears to be no data available to support this and clopidogrel is not currently licensed for use in DVT prophylaxis.

In our survey 13.9% of respondents did not routinely stop clopidogrel pre-operatively. None of these respondents reported any increased incidence of pre-operative or post-operative complications. This anecdotally seems to suggest that continued use of clopidogrel in patients undergoing orthopaedic surgery may not result in increased risk of complications such as bleeding. This is however contrasted by the reports of increased peri-operative complications (namely bleeding) in those who stop the medication pre-operatively. The finding of increased complications attributable to clopidogrel in those who stop the medication compared with those who do not is paradoxical. There is no literature available to suggest a rebound coagulopathy in patients' that stop clopidogrel pre-operatively. Indeed the likely alternative explanation is that as assessment of bleeding is subjective, consultants who are aware of the complications of clopidogrel may attribute any bleeding to the medication despite having stopped it for a period greater than its' supposed physiologic effect; and those who do not stop the medication may not attribute any increased complications to it. This may be due to a lack of understanding of the mechanism of action of the drug and its effects. There is also the possibility that those who continue clopidogrel are more meticulous with haemostasis due to knowledge of the potential for increased complications. However we are unable to draw any firm conclusions due to the small sample size of our survey.

Our study indicates that there is a lack of uniformity amongst orthopaedic surgeons, and haematologists in Scotland, with regards the need to stop clopidogrel preoperatively. There also appears to be some confusion as to whether additional DVT prophylaxis is needed. Most orthopaedic units did not have a policy to deal with these patients, and the majority did not contact the haematology department at their hospital prior to instituting a policy on stopping clopidogrel. The majority of orthopaedic surgeons also felt that further guidelines in this area would be useful. In order to best manage these patients a multidisciplinary approach is needed to fully assess risk of bleeding compared to risk of an ischemic event when assessing need for anticoagulation.

Our survey raises the following issues. First, is it safe to continue taking clopidogrel in the perioperative period, and if so, is any additional DVT prophylaxis needed or is clopidogrel alone sufficient? Second, in the absence of any current guidelines, are there medico-legal implications if surgeons fail to conform to the British National Formulary advice of stopping clopidogrel 7 days preoperatively? Third, should elective/emergency surgery be delayed until the antiplatelet effect of clopidogrel has worn off?

As this paper was an observational study, there are limitations. Although more than 60% of consultants responded there is the potential for bias as those who responded may have more interest in this subject perhaps due to knowledge of the medication or previous experience of complications. There is also the possibility that those who did not respond do not stop the medication and experienced complications. This would alter the results and therefore the conclusions drawn. However the purpose of this study was to audit the current practice in Scotland and highlight a potential lack of uniformity and the need for further guidance on peri-operative management of these patients.

## Conclusion

This is an area of orthopaedic practice that requires further research, as there is great variation in practice. At present it appears that patients having elective surgery should ideally stop clopidogrel 7 days in advance of surgery and that if additional DVT prophylaxis is needed a LMWH or fondaparinux should be used. This should only be done once the patients' condition has been discussed with a haematologist and/or a cardiologist and the benefit/risk of anticoagulation considered. In the emergency setting (trauma), patients need to be assessed on a case-by-case basis. In situations where the risk of intra-operative bleeding in patients taking clopidogrel is less than the morbidity/mortality of delaying treatment, (e.g. fractured neck of femur [16-18]), surgery should not be delayed. Surgeons should be aware of the mechanism of action of clopidogrel and its potential side effects, and surgical technique should be modified accordingly.

## Competing interests

The author(s) declare that they have no competing interests.

## Authors' contributions

JJJ and AP conceived of the study and participated in its design. JJJ was the first author and wrote the article in conjunction with AP. DB was the supervising Consultant.

All authors read and approved the final manuscript.

## Supplementary Material

Additional File 1 **Raw Database**. Clopidogrel audit.mdb – Database viewable in Microsoft access. Click here for file
